# EP300 Modulates MCM8 Transcription and Augments the Malignant Phenotype of Hepatitis B Virus–Positive Hepatocellular Carcinoma Cells

**DOI:** 10.1002/kjm2.70006

**Published:** 2025-03-17

**Authors:** Fang Xue, Tian‐Feng Sun

**Affiliations:** ^1^ Department of Liver Disease Infection Suzhou Hospital of Integrated Traditional Chinese and Western Medicine Suzhou Jiangsu People's Republic of China

**Keywords:** acetylation modification, EP300, hepatitis B virus, hepatocellular carcinoma, MCM8

## Abstract

Chronic infection with the hepatitis B virus (HBV) remains one of the primary drivers of the development of hepatocellular carcinoma (HCC), a highly aggressive malignancy with a grim prognosis. This study focused on the role of E1A‐binding protein p300 (EP300) in the malignant phenotype of HBV‐positive HCC cells and its functional mechanism. Increased EP300 expression was detected in HBV‐positive tumor tissues and cells compared to their control counterparts. Silencing EP300 reduced tumorigenic activity, proliferation, viability, migration, invasion, and resistance to apoptosis of HBV‐positive cells and reduced the concentrations of HBV infection markers HBsAg and HBeAg. These effects were achieved, at least in part, through downregulation of minichromosome maintenance 8 homologous recombination repair factor (MCM8). *MCM8* was identified as a target of EP300 and mediated by acetylation modification. MCM8 was upregulated in HBV‐positive tumors and HCC cells while decreasing following EP300 silencing in cells. However, the restoration of *MCM8* expression in these cells rescued their malignant properties. In summary, this study suggests a role for EP300‐mediated *MCM8* upregulation in the malignant properties of HBV‐positive HCC.

## 1 Introduction

In 2022, liver cancer was responsible for over 750,000 deaths globally, representing the third leading cause of cancer‐related death and the sixth most prevalent cancer worldwide [1]. China alone is responsible for over half of these new cases and deaths, with approximately 431,000 new incidences and 412,000 deaths in 2022 [2]. Hepatocellular carcinoma (HCC), which develops from hepatocytes, the main parenchymal cells of the liver, is the most prevalent subtype, constituting over 80% of primary liver cancer cases [3]. Despite advancements in treatment options, such as surgical resection, liver transplantation, radiation, percutaneous ablation, and transarterial and systemic therapies [4], the prognosis of HCC remains poor [5]. This severe condition requires a deeper understanding of its pathogenesis and the development of new therapeutic strategies.

Chronic hepatitis B virus (HBV) infection is a common but underdiagnosed and undertreated health condition and is the leading cause of HCC worldwide [6]. Later research identified several mechanisms through which HBV infection can lead to the malignant transformation of hepatocytes, such as HBV gene integration, mutation‐induced genomic instability, and tumor‐driving signaling pathway activation [7]. Epigenetic regulation has emerged as a significant factor in the activation of oncogenic pathways modulated by HBV proteins [8]. Histone acetylation, which is associated with gene transcription, is performed by three types of histone acetyltransferases belonging to the GNAT, MYST, and CBP/P300 families [9]. The E1A‐binding protein p300 (EP300), also referred to as P300, serves as a crucial regulator of chromatin and functions as an oncogenic transcription factor [10]. In addition, it is responsible for the acetylation of histone H3 lysine 18 (H3K18ac) and H3K27ac modifications associated with active promoters and enhancers [11]. For instance, silencing of EP300 significantly suppressed the interaction between H3K27ac and the SLC38A6 promoter to decrease SLC38A6 expression, thereby controlling glutamine metabolism and mitochondrial respiration in HCC [12]. Increased EP300 expression has been linked to aggressive HCC characteristics and poor prognosis [13]. However, the detailed mechanism of H3K27ac modification by EP300 in HBV‐positive HCC remains to be further explored. The minichromosome maintenance family (MCMs), a replication licensing factor, is involved in the pathogenesis of tumors, and MCM8 was one of the most upregulated members of this family in HCC (2.79‐fold relative to noncancerous liver) [14]. Each MCM subunit has a distinct function achieved by differential post‐translational modifications in both the DNA replication process and the response to replication stress [15]. Given these insights, this study aimed to analyze EP300's function in HBV‐positive HCC development and the underlying mechanisms involving MCM8.

## 2 Materials and Methods

### 2.1 Clinical Specimen Collection

Tumor and adjacent non‐involved tissues were collected from 24 HBV‐positive HCC patients treated at the Suzhou Hospital of Integrated Traditional Chinese and Western Medicine from April 2021 to April 2024. Tumor and adjacent liver tissues were surgically collected and embedded into paraffin‐embedded pathological specimens. The inclusion criteria were as follows: (1) HCC confirmed by pathological examination due to positive serum hepatitis B surface antigen (HBsAg); (2) patients who had undergone radical liver resection and had never received HBV antiviral therapy before radical liver resection; and (3) none of them had hepatitis C, nonalcoholic fatty liver disease, or alcoholic liver disease. The clinicopathological characteristics of the patients are presented in Table S1. Informed consent was obtained from all patients. All human experiments were approved by the Ethics Review Committee of Suzhou Hospital of Integrated Traditional Chinese and Western Medicine and were performed in strict adherence to the *Declaration of Helsinki*.

### 2.2 Cells and Treatment

The human liver epithelial cell line THLE‐2 (CRL‐2706) and two HBV‐positive HCC cell lines, SNU‐398 (CRL‐2233) and Hep3B (HB‐8064) were obtained from ATCC (Manassas, VA, USA). The cells were cultured at 37°C with 5% CO_2_ in DMEM supplemented with 10% fetal bovine serum (FBS) and 1% antibiotics.

Hep3B and SNU‐398 cells were transfected with the HBV genome‐containing pGEMHBV (Miaolingbio.Inc., Wuhan, Hubei, China) by Lipofectamine 3000 transfection reagent (L3000001, Invitrogen Inc., Carlsbad, CA, USA). Lentiviral vectors for EP300 knockdown (KD‐EP300), MCM8 overexpression (LV‐MCM8), and negative control (NC) vectors (KD‐NC and LV‐NC) were provided by GeneCopoeia Inc. (Rockville, MD, USA). The lentiviral vector solution (> 10^7^ TU/mL) was introduced into Hep3B and SNU‐398 cells, and stably infected (transfected) cells were selected with puromycin for 2 weeks.

### 2.3 Analysis of Tumorigenic Activity

BALB/c nude mice (5 weeks old, weighing 17.5 g, Vital River, Beijing, China) were kept under appropriate conditions. The mice were allocated to four groups: KD‐NC, KD‐EP300, KD‐EP300 + LV‐NC, and KD‐EP300 + LV‐MCM8, each consisting of five mice. Stably transfected SNU‐398 cells were digested, washed, and resuspended at a density of 1 × 10^7^ cells/mL. An equal volume (0.1 mL) was injected subcutaneously into each mouse. The tumor length (*L*) and width (*W*) were determined using a caliper. Tumor volume (*V*) was calculated using the formula *L* × *W* ^2^ × 0.5. Four weeks postinjection, the mice were euthanized by intraperitoneal administration of nembutal (150 mg/kg). Tumor tissues were extracted, weighed, and prepared for further analysis. Animal experiments were approved by the Animal Ethics Committee of Suzhou Hospital of Integrated Traditional Chinese and Western Medicine and conducted following the Guide for the Care and Use of Laboratory Animals (NIH, Bethesda, MD, USA).

### 2.4 mRNA Expression Analysis

Total RNA was isolated from clinical specimens and HCC cells using TRIzol reagent (10296010CN, Thermo Fisher Scientific). The isolated RNA was reverse‐transcribed into cDNA using EasyScript First‐Strand cDNA Synthesis SuperMix (AE301‐02; TransGen Biotech, Beijing, China). qPCR was performed using the TB Green Premix Ex Taq II kit (RR820Q; TaKaRa) on an ABI7500 real‐time PCR instrument (ABI Company, Oyster Bay, NY, USA). With GAPDH serving as the reference gene, relative expression was calculated using the 2^−∆∆Ct^ method. The primers used included: *EP300* (F) 5′‐GATGACCCTTCCCAGCCTCAAA‐3′, *EP300* (R) 5′‐GCCAGATGATCTCATGGTGAAGG‐3′; *MCM8* (F) 5′‐CTGTGTGTCGAGGCAGGTCATT‐3′, *MCM8* (R) 5′‐TCGTGGAATCCGACCTGCTTCT‐3′; *GAPDH* (F) 5′‐GTCTCCTCTGACTTCAACAGCG‐3′, *GAPDH* (R) 5′‐ACCACCCTGTTGCTGTAGCCAA‐3′.

### 2.5 Protein Expression Analysis In Vitro

The HCC cell lines were lysed using RIPA lysis buffer (P0013E, Beyotime), and the total protein content was evaluated using the Pierce BCA Protein Assay Kit (A65453, Thermo Fisher Scientific). The protein samples were separated by 10% SDS‐PAGE and loaded onto polyvinylidene fluoride membranes (ab133411, Abcam Inc., Cambridge, MA, USA). After blocking with 5% skimmed milk for 1 h, the membranes were probed with EP300 (1:1000, ab275378, Abcam) and GAPDH (1:10,000, AC001, ABclonal Technology Co. Ltd., Wuhan, Hubei, China) antibodies overnight at 4°C. After thorough washing, the membranes were incubated with goat anti‐rabbit IgG H&L (1:2000, AS014, ABclonal) for 1 h. The signal bands were developed using BeyoECL Plus (P0018S; Beyotime). Relative protein levels, normalized to GAPDH, were evaluated using the ImageJ software.

### 2.6 Protein Expression in Tumor Tissues

Human and mouse tumor tissues were fixed and paraffinized to prepare tissue sections. Following deparaffinization and rehydration, the sections were placed in 0.01 M citrate buffer and heated for 20 min for antigen retrieval. After treatment with 3% H_2_O_2_ and blocking with 5% goat serum (C0265, Beyotime), the sections were probed with primary antibodies against EP300 (1:200, A13016, ABclonal), MCM8 (AV36668, Sigma‐Aldrich, Merck KGaA, Darmstadt, Germany), and Ki67 (1:100, A20018, ABclonal) overnight at 4°C, followed by incubation with goat anti‐rabbit IgG H&L (1:200, AS014, ABclonal) for 30 min. After color development using DAB (P0203, Beyotime), the sections were counterstained with hematoxylin (C0107, Beyotime). Positive staining was evaluated via microscopic observation.

### 2.7 HBsAg and HBeAg Measurement

The concentrations of HBsAg and HBeAg in cell culture supernatants or xenograft tumors were determined using HBsAg (ABIN6962773, Antibodies‐online GmbH, Aachen, Germany) and HBeAg (EHJ‐92825h, Huijia Biotechnology Co. Ltd., Xiamen, Fujian, China) ELISA Kits. Briefly, the supernatants from the cell culture were centrifuged at 1000*g* for 20 min at 4°C to remove impurities and cell debris. Xenograft tumor tissues were lysed and centrifuged at 3000*g* for 10 min. The supernatants were diluted and added to the wells of an ELISA plate. The plate was covered and incubated for 90 min at 37°C. This was followed by the addition of 100 μL of biotinylated antibody working solution for 1 h and 100 μL of HRP‐conjugated working solution for 30 min. Subsequently, 90 μL tetramethylbenzidine substrate solution was added and incubated in the dark at 37°C for 15 min. After the final addition of the stop solution, the optical density at 450 nm was measured using a microplate reader.

### 2.8 Analysis of HCC Cell Viability

Following the protocol of the EdU Assay Kit (ab222421, Abcam), the cells were cultured in 24‐well plates (5 × 10^4^ cells per well) for 24 h. After a 2‐h incubation with EdU working solution, the cells were fixed, permeabilized, and incubated with Apollo staining solution for 30 min. Edu‐labeled cells were captured and counted using a microscope.

### 2.9 Analysis of HCC Cell Proliferation

Cell viability was assessed using the CCK‐8 Kit (C0037, Beyotime). Cell suspensions from each group were seeded in 96‐well plates (100 μL/well). After 24 h of incubation, 10 μL of the CCK‐8 solution was added to each well. Following a 15‐min incubation, the optical density was measured at 450 nm to evaluate cell viability.

The prepared HCC cells were digested, centrifuged, and resuspended in 1 × 10^3^ cells/mL. The cells were then plated into six‐well plates at 500 cells/well and cultured for 2 weeks. The medium was refreshed every 3 d. Following the 2‐week incubation, the cells were fixed and stained with 0.1% crystal violet (C0121, Beyotime) for 20 min. After air drying, the number of cell colonies was determined using a microscope.

### 2.10 Analysis of HCC Cell Migration and Invasion

Transwell chambers with an 8 μm pore diameter were used to analyze cell migration and invasion. The upper chambers were pre‐coated with Matrigel, specifically for invasion assays. The HCC cells were plated into the apical chambers filled with a serum‐free medium at a density of 1 × 10^5^ cells per well, while the basolateral chambers were filled with a complete medium containing 10% serum as a chemoattractant. After incubation at 37°C for 24 h, the migrating or invading cells on the underside of the membranes were fixed, stained with 0.1% crystal violet, and counted under a microscope.

### 2.11 Apoptosis Assay

The cells were stained using the Click‐iT Plus TUNEL Assay Kit (C10617, Invitrogen). Briefly, HCC cells were seeded into 24‐well plates with glass coverslips and incubated for 48 h. The cells were then fixed, permeabilized, and blocked with 1% bovine serum albumin (37,525, Thermo Fisher Scientific). Nuclei were stained with DAPI (C1002, Beyotime). Images were captured using an inverted microscope camera system. Apoptotic cells appeared as green‐stained cells under fluorescence microscopy.

### 2.12 Evaluation of Binding of EP300 and the *MCM8* Promoter

Following the protocol of the Pierce Magnetic ChIP Kit (26,157, Thermo Fisher Scientific), HCC cell lines from each group were treated with 1% formaldehyde for DNA‐protein crosslinking. The cells were then lysed and subjected to ultrasonication to truncate the chromatin to 200–300 bp fragments. The samples were incubated with antibodies against EP300 (1:200, ab275378, Abcam), H3K27ac (1:10, A7253, ABclonal), and IgG (1:1000, ab172730, Abcam) overnight at 4°C. Protein A/G magnetic beads were used to capture the immunoprecipitated complexes. The complexes were de‐crosslinked. Subsequently, the DNA was eluted and purified, and the abundance of the *MCM8* promoter fragment was analyzed using qPCR.

### 2.13 Statistical Analysis

Measurement data were collected from a minimum of three biological replicates and are expressed as mean ± standard error of the mean. Data were analyzed using Prism software (version 8.0.2) (GraphPad, La Jolla, CA, USA). Differences were compared using the Student's *t*‐test or by one‐ or two‐way analysis of variance (ANOVA) plus Tukey's multiple comparisons, as appropriate. Statistical significance was defined as a *p*‐value of < 0.05.

## 3 Results

### 3.1 EP300 Exhibits Heightened Expression in HBV‐Positive HCC

**FIGURE 1:**
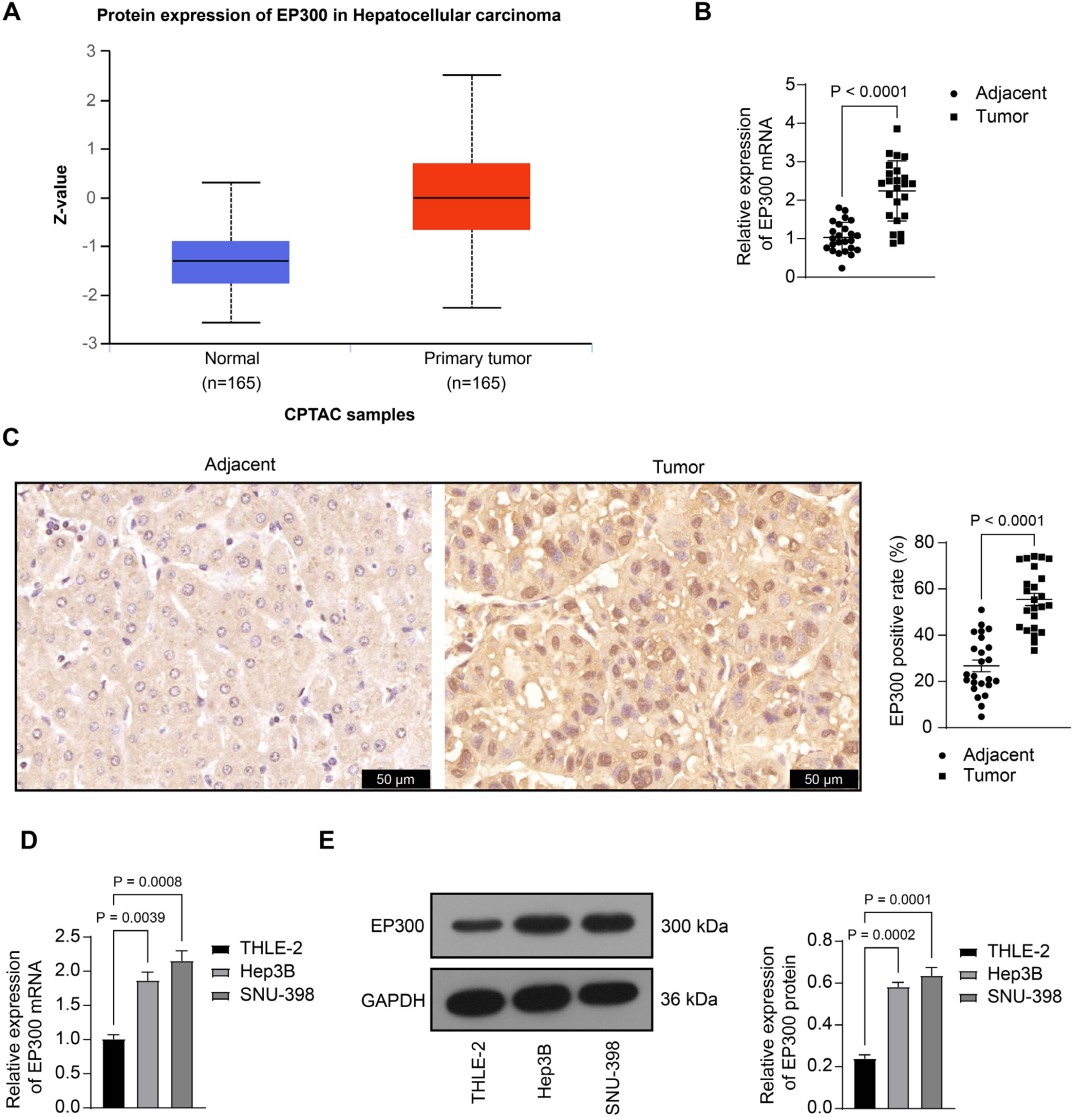
EP300 exhibits heightened expression in HBV‐associated HCC. (A) EP300 protein level in HCC and normal cohorts according to the UALCAN data; (B) mRNA expression of EP300 in tumor and adjacent non‐involved tissues from patients with HBV‐positive HCC (*n* = 24) determined using RT‐qPCR; (C) positive staining of EP300 in the clinical tissue samples (*n* = 24) determined using IHC; (D, E) mRNA (D) and protein (E) levels of EP300 in THLE‐2, SNU‐398, and Hep3B cells determined using RT‐qPCR and WB analysis, respectively. For cellular experiments, three biological replicates were performed. Differences were compared by the paired *t*‐test (B, C) or one‐way ANOVA (D, E).

As a histone acetyltransferase and transcriptional coactivator, EP300 is correlated with an aggressive phenotype and poor prognosis in HCC [13]. Data from the UALCAN system (https://ualcan.path.uab.edu/index.html) also revealed increased EP300 protein levels in HCC patients compared to normal cohorts (Figure 1A). To investigate the correlation between EP300 and HBV‐associated HCC, we harvested tumor and adjacent non‐involved tissues from patients with HBV‐positive HCC. Notably, increased mRNA expression (Figure 1B) and positive staining (Figure 1C) of EP300 were determined in the tumor tissues.

Furthermore, a human liver epithelial cell line THLE‐2 and two HCC cell lines SNU‐398 and Hep3B pre‐infected with HBV genomic DNA were used for in vitro experiments. Notably, compared to THLE‐2 cells, SNU‐398 and Hep3B cells exhibited significantly heightened mRNA and protein levels of EP300 (Figure 1D,E).

### 3.2 *EP300* Silencing Reduced Tumorigenesis of SNU‐398 Cells in Nude Mice

**FIGURE 2:**
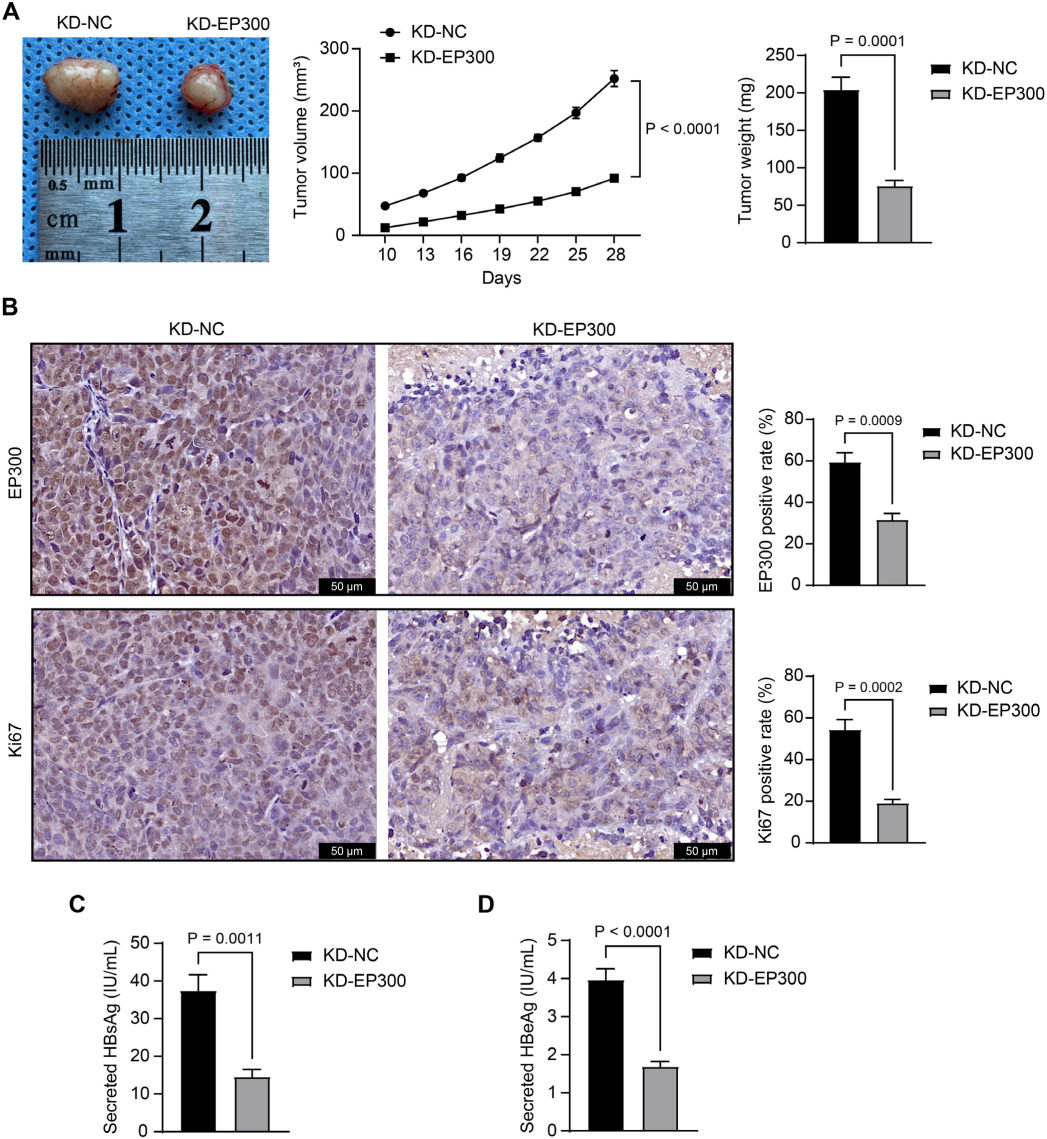
*EP300* silencing reduced tumorigenesis of SNU‐398 cells in nude mice. SNU‐398 cells stably transfected with KD‐NC or KD‐EP300 were implanted into the mice subcutaneously. (A) The volume and weight of the xenograft tumors; (B) The positive rate of EP300 and Ki67 in the tumor tissues was examined using IHC; (C, D) concentrations of HBsAg (C) and HBeAg (D) in xenograft tumors determined using ELISA kits. Each group contained five mice. Differences were compared by the unpaired *t*‐test (A–D).

To reduce animal use, only the SNU‐398 cell line, which presented the highest EP300 level, was used for in vivo experiments. SNU‐398 cells stably transfected with KD‐NC or KD‐EP300 were subcutaneously implanted into mice. Cells with KD‐EP300 showed significantly reduced tumorigenic activity, which was marked by decreased tumor volume and weight (Figure 2A). The IHC assay suggested decreased EP300 staining in the xenograft tumors formed by KD‐EP300‐transfected cells, accompanied by reduced Ki67 staining (Figure 2B). Additionally, the ELISA results demonstrated a decrease in the concentrations of HBsAg (Figure 2C) and HBeAg (Figure 2D) in xenograft tumors upon EP300 knockdown.

### 3.3 EP300 Silencing Curbs Malignant Phenotype of HBV‐Positive HCC Cells In Vitro

**FIGURE 3:**
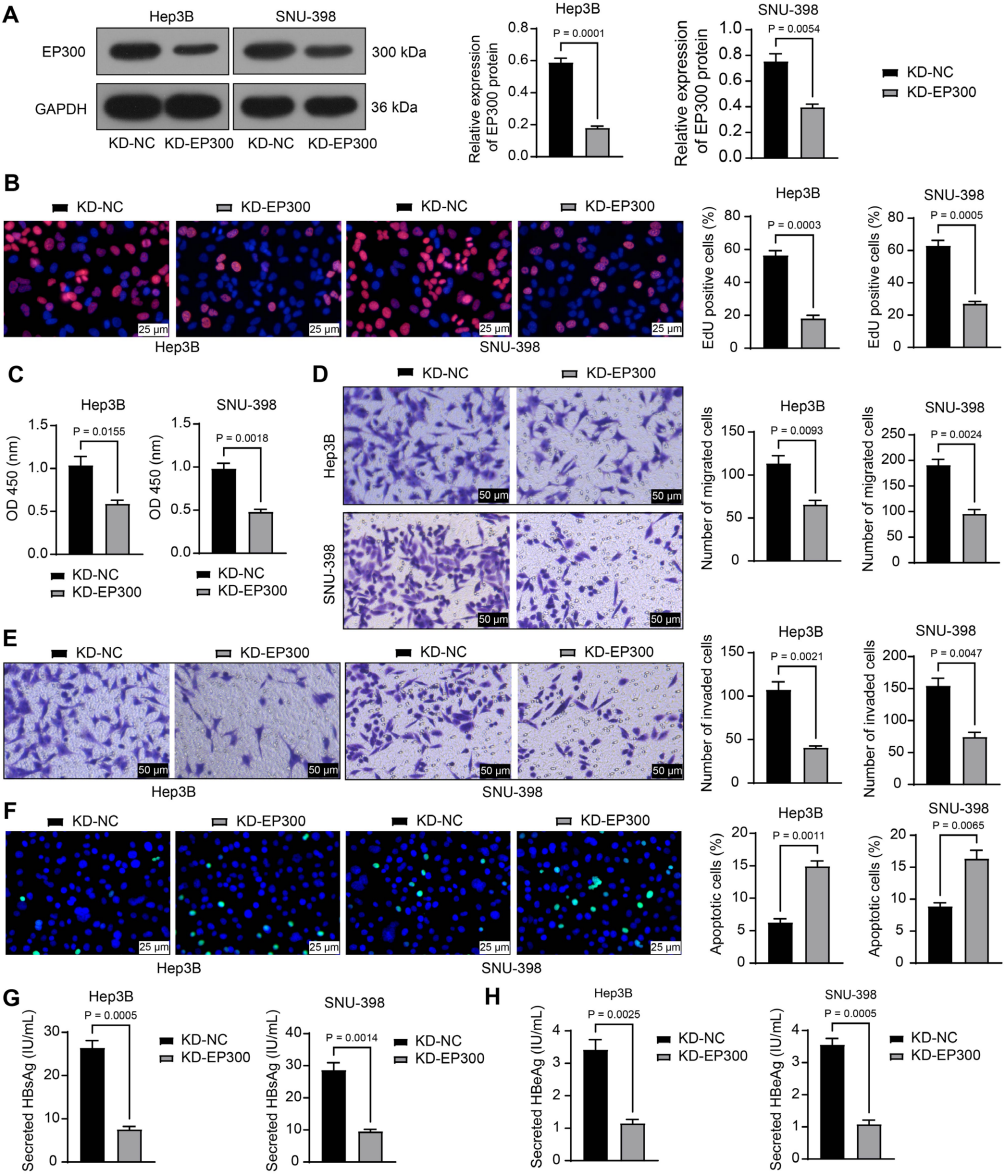
EP300 silencing inhibits the malignant behavior of HBV‐positive HCC cells in vitro. SNU‐398 and Hep3B cells were stably transfected with KD‐NC or KD‐EP300. (A) Protein level of EP300 in cells determined using WB analysis; (B) proliferation of cells determined using EdU labeling assay; (C) viability of cells analyzed by CCK‐8 assay; (D, E) migration (D) and invasion (E) capacities of cells analyzed by Transwell assays; (F) apoptosis in cells determined using TUNEL assay; (G, H) concentrations of HBsAg (G) and HBeAg (H) in cell culture supernatant determined using ELISA kits. Three biological replicates were performed. Differences were compared by the unpaired *t*‐test (A–H).

To analyze the role of EP300 in HBV‐positive HCC cells, SNU‐398 and Hep3B cells were transfected with KD‐NC or KD‐EP300. KD‐EP300 administration significantly reduced the protein levels of EP300 in both cell lines (Figure 3A). This led to substantially reduced proliferation and viability of the cells according to EdU labeling (Figure 3B) and CCK‐8 assays (Figure 3C). Furthermore, Transwell assays demonstrated a decrease in the migration and invasion abilities of SNU‐398 and Hep3B cells following *EP300* knockdown (Figure 3D,E). In contrast, apoptosis in these cells, according to the TUNEL assay (Figure 3F), significantly increased upon *EP300* silencing. Mirroring the in vitro findings, reduced concentrations of HBsAg (Figure 3G) and HBeAg (Figure 3H) were detected in the cell culture supernatant upon *EP300* silencing.

### 3.4 EP300 Enhances MCM8 Expression Through Acetylation Modification

**FIGURE 4:**
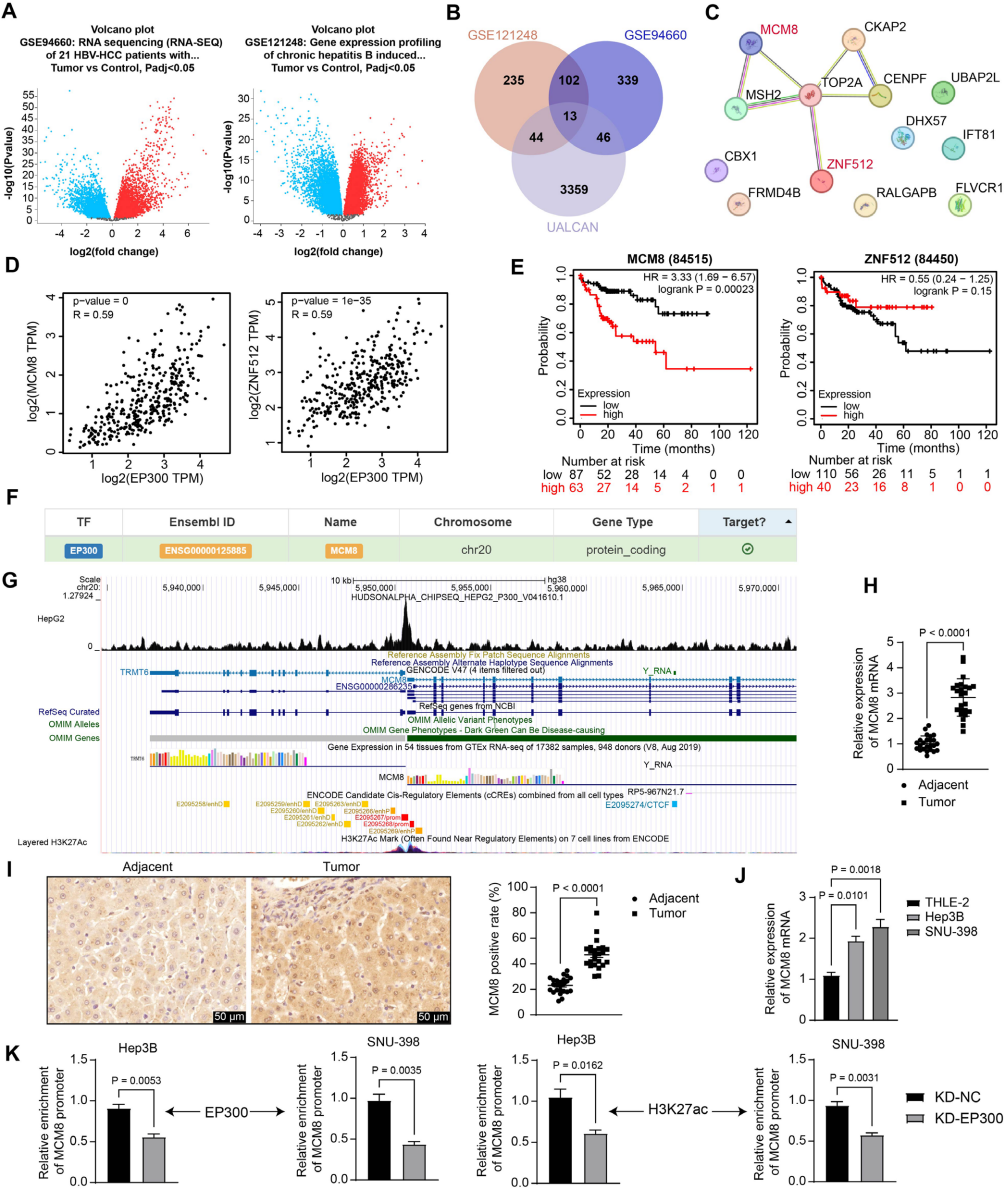
EP300 increases MCM8 expression through acetylation modification. (A) Volcano plots for DEGs between HBV‐positive HCC tumors and the non‐tumor tissues identified using GSE94660 and GSE121248 datasets; (B) intersection analysis of significant DEGs and the top 500 genes positively correlated with EP300 (Pearson correlation coefficient > 0.5) in HCC predicted from the UALCAN system; (C) a protein–protein interaction analysis of the intersecting genes; (D) positive correlations between EP300 and MCM8 or ZNF512 in HCC predicted using the GEPIA system; (E) prognostic values of MCM8 and ZNF512 in HBV‐positive HCC patients predicted from the Kaplan–Meier Plotter system; (F) a putative interaction between EP300 and MCM8 suggested by the hTFtarget system; (G) existence of binding peaks of EP300 and H3K27ac near the *MCM8* promoter region according to the ChIP‐seq data; (H) mRNA expression of *MCM8* in tumor and adjacent non‐involved tissues from patients with HBV‐positive HCC (*n* = 24) determined using RT‐qPCR; (I) positive staining of MCM8 in the clinical tissue samples (*n* = 24) determined using IHC; (J) mRNA expression of *MCM8* in THLE‐2, SNU‐398, and Hep3B cells determined using RT‐qPCR; (K) binding of EP300 and H3K27ac to the *MCM8* promoter in SNU‐398 and Hep3B cells transfected with KD‐NC or KD‐EP300 determined using ChIP‐qPCR assay. For cellular experiments, three biological replicates were performed. Differences were compared by the paired *t*‐test (H, I), the unpaired *t*‐test (K), or one‐way ANOVA (J).

To explore the downstream molecules affected by EP300 implicated in the aforementioned results, we explored the genes showing positive correlations with EP300 (Pearson correlation coefficient > 0.5) in HCC using the UALCAN system. Moreover, two GEO datasets, GSE94660 and GSE121248, were employed to identify differentially expressed genes (DEGs) between HBV‐positive HCC tumors and non‐tumor tissues, with significant DEGs identified by an adjusted *p*‐value < 0.05 (Figure 4A). These DEGs intersected with the top 500 genes positively correlated with EP300, leading to the identification of 13 intersections: TOP2A, MSH2, DHX57, FLVCR1, CKAP2, UBAP2L, ZNF512, MCM8, CENPF, IFT81, RALGAPB, FRMD4B, and CBX1 (Figure 4B). Protein interaction analysis revealed interactions between MCM8, MSH2, TOP2A, CKAP2, CENPF, and ZNF512 (Figure 4C). Among these, MSH2 [16], TOP2A [17], CKAP2 [18], and CENPF [19] have been associated with the malignant properties of HCC cells, whereas MCM8 and ZNF512 are the only two factors whose roles in HCC development remain unknown. According to data from the GEPIA system (http://gepia.cancer‐pku.cn/index.html), EP300 was positively correlated with both MCM8 and ZNF512 in HCC (Figure 4D), with a greater correlation between EP300 and MCM8. Furthermore, data from the Kaplan–Meier Plotter system (https://kmplot.com/analysis/) revealed that MCM8 was associated with poor prognosis in HBV‐positive HCC patients, while ZNF512 did not present a statistically significant correlation with patient prognosis (Figure 4E). Therefore, we selected MCM8 for further investigation. Interestingly, the data from both the GSE94660 and GSE121248 datasets revealed upregulation of EP300 and MCM8 in HBV‐positive HCC tumors compared to non‐tumor tissues (LogFC > 0). The hTFtarget system (https://guolab.wchscu.cn/hTFtarget/#!/) revealed a putative interaction between EP300 and MCM8 (Figure 4F). ChIP‐seq data (http://cistrome.org/db/#/) suggested significant binding peaks of EP300, and the acetylation modification marker H3K27ac was predicted near the *MCM8* promoter region (Figure 4G). Therefore, we speculated that EP300 modulates *MCM8* transcription through acetylation to affect the progression of HBV‐positive HCC.

Notably, examination of the clinical samples revealed increased mRNA expression (Figure 4H) and positive staining (Figure 4I) of MCM8 in the tumor tissues compared to the normal tissues. Additionally, increased mRNA expression of *MCM8* was detected in SNU‐398 and Hep3B cells compared to that in normal THLE‐2 cells (Figure 4J). Furthermore, ChIP‐qPCR assays revealed that the binding between EP300 or H3K27ac and the *MCM8* promoter in SNU‐398 and Hep3B cells decreased upon EP300 silencing (Figure 4K). This indicates that EP300 binds to the *MCM8* promoter and enhances its expression in HBV‐positive HCC cells.

### 3.5 Overexpression of *MCM8* Restores Tumorigenic Activity of SNU‐398 Cells in Nude Mice

**FIGURE 5:**
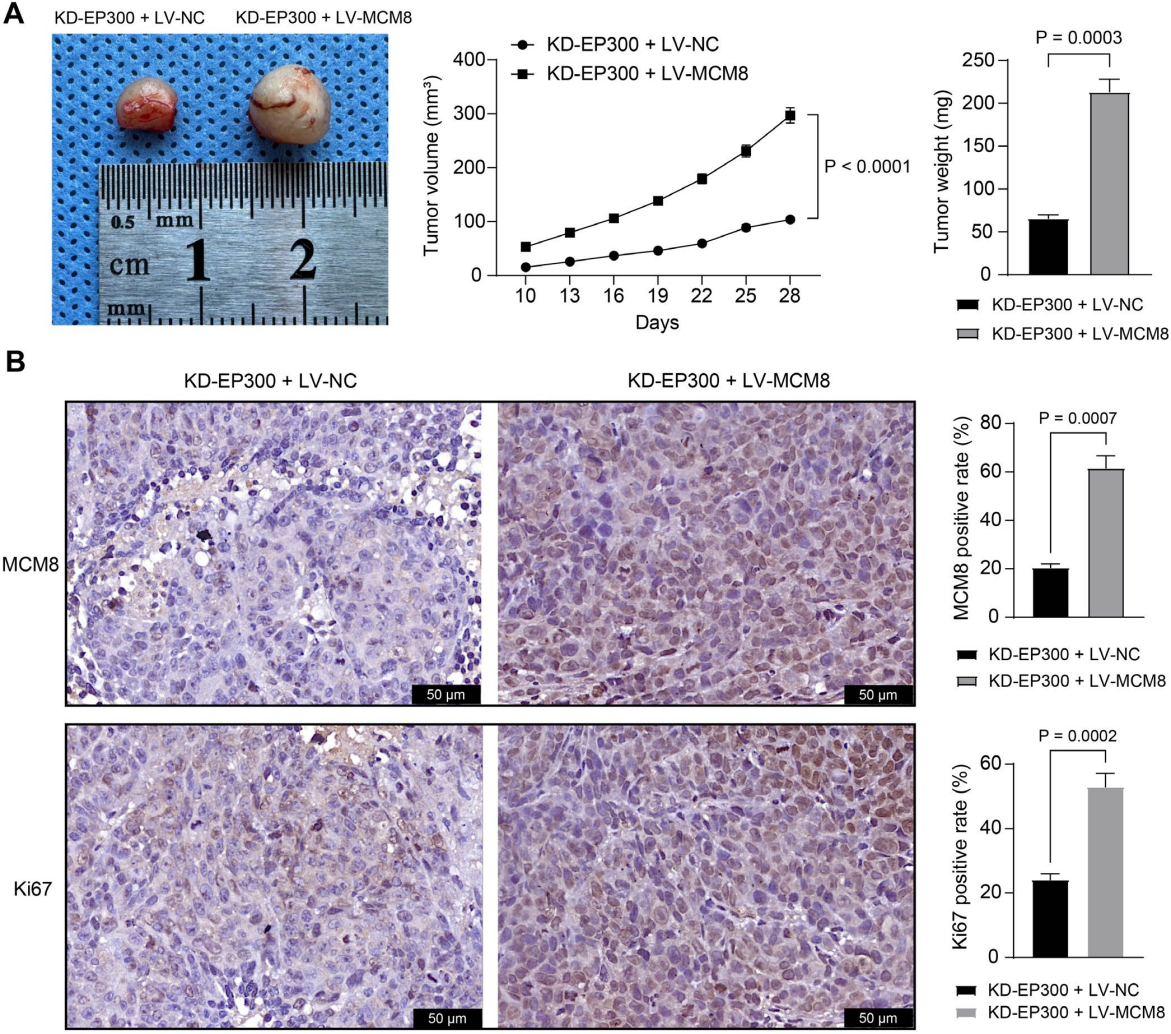
Overexpression of MCM8 restores tumorigenesis of SNU‐398 cells in nude mice. SNU‐398 cells stably administered KD‐EP300 were additionally transfected with LV‐MCM8 or LV‐NC, followed by subcutaneous injection into nude mice. (A) The volume and weight of the xenograft tumors; (B) the positive rate of MCM8 and Ki67 in the tumor tissues was examined using IHC. Each group contained five mice. Differences were compared by the unpaired *t‐*test (A, B).

SNU‐398 cells stably administered KD‐EP300 were additionally transfected with LV‐MCM8 or LV‐NC, which were then subcutaneously injected into nude mice. Notably, the volume and weight of xenograft tumors, initially curbed by EP300 silencing, were significantly restored following MCM8 overexpression (Figure 5A,B).

### 3.6 Overexpression of MCM8 Enhances the Malignant Phenotype of HBV‐Positive HCC Cells In Vitro

**FIGURE 6:**
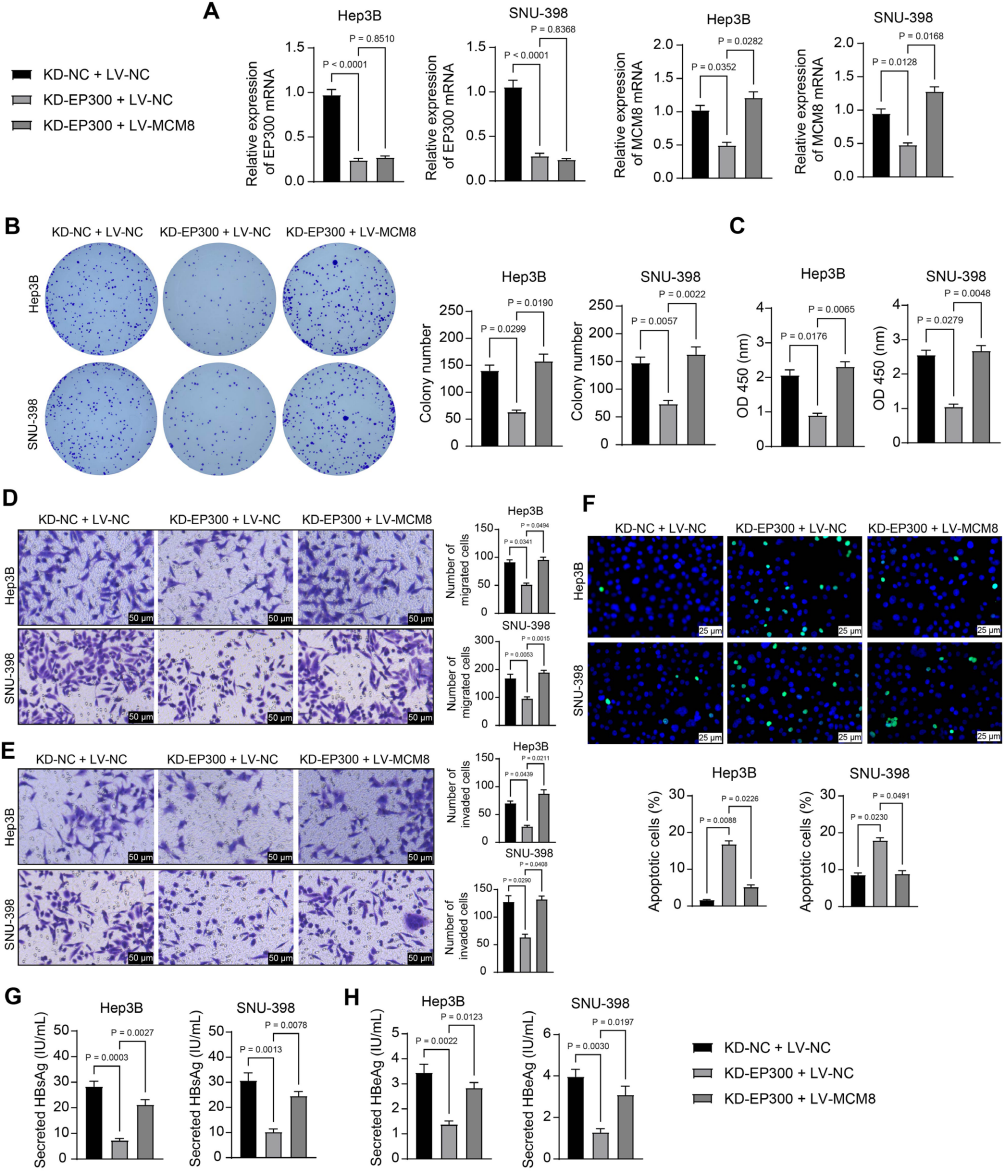
Overexpression of MCM8 restores the malignant phenotype of HBV‐positive HCC cells suppressed by KD‐EP300 in vitro. SNU‐398 and Hep3B cells were stably infected with KD‐NC + LV‐NC, KD‐EP300 + LV‐NC, or KD‐EP300 + LV‐MCM8. (A) mRNA expression of *EP300* and *MCM8* in cells determined using RT‐qPCR; (B) colony formation ability of cells determined using a colony formation assay; (C) viability of cells analyzed by a CCK‐8 assay; (D, E) migration (D) and invasion (E) capacities of cells analyzed by Transwell assays; (F) apoptosis in cells determined using a TUNEL assay. (G, H) Concentrations of HBsAg (G) and HBeAg (H) in cell culture supernatants determined using ELISA kits. Three biological replicates were performed. Differences were compared by the one‐way ANOVA (A–H).

KD‐NC + LV‐NC, KD‐EP300 + LV‐NC, and KD‐EP300 + LV‐MCM8 were introduced into SNU‐398 and Hep3B cells. RT‐qPCR showed that compared with the KD‐NC + LV‐NC group, KD‐EP300 treatment downregulated the mRNA levels of both *EP300* and *MCM8*, whereas LV‐MCM8 only upregulated *MCM8* mRNA levels without affecting the expression levels of *EP300* (Figure 6A). Importantly, the colony formation assay revealed that *MCM8* upregulation significantly promoted the colony formation ability of the two cell lines in the presence of *EP300* knockdown (Figure 6B). The *EP300* knockdown–mediated inhibition of cell viability, migration, and invasion was diminished by *MCM8* overexpression (Figure 6C–E). Furthermore, apoptosis in SNU‐398 and Hep3B cells promoted by *EP300* silencing was reduced following additional *MCM8* overexpression (Figure 6F). Finally, ELISA results showed that MCM8 overexpression reversed the downregulation of HBsAg and HBeAg levels by KD‐EP300 (Figure 6G,H).

## 4 Discussion

HCC development is a complex process involving dysregulated cellular and molecular events driven by genetic and epigenetic alterations [20]. For instance, we have previously shown that ETV4 mediates transcriptional activation of ANXA2 to promote HBV‐associated HCC progression [21]. HBV vaccination or antiviral therapy can reduce the risk of HCC, but they do not eliminate it [22]. This study identified EP300 as a key molecule that is associated with the malignant phenotype of HBV‐related HCC cells. Further investigation revealed that MCM8 is a critical contributor to EP300‐mediated oncogenic events in this context.

EP300 has been revealed by Ring et al. to enhance the cancer stem cell phenotype, thereby representing a potential target to address tumor initiation and metastatic spread in triple‐negative breast cancer [23]. Elevated EP300 expression has been identified in HCC tumors, which are associated with larger tumor size, poorer differentiation, and more advanced stages, making EP300 a promising independent prognostic factor [13]. Additionally, increased EP300 expression leads to the acetylation of myocyte enhancer factor 2D, which binds to the promoter region of the CD274 gene (encoding programmed cell death‐ligand 1) and enhances its transcription [24]. Silencing EP300 reduces the H3K27ac modification near the promoter region of solute carrier family 38 member 6, a factor involved in glutamine metabolism, mitochondrial respiration viability, cell cycle progression, and colony formation in HCC cells [12]. Tropberger et al. demonstrated that a small molecule inhibitor of EP300 reduced HBV transcription in a dose‐dependent manner [25]. Molecular docking analysis by Xu et al. identified EP300 as a target of several components of the Ganweikang tablet, a Traditional Chinese Medicine used to treat chronic hepatitis B [26]. Notably, increased EP300 expression was detected in HBV‐positive HCC tumor tissues and cells compared to control counterparts. Ki‐67 is one of the most famous markers used to identify proliferating cells [27]. EP300 overexpression enhanced the protein expression of Ki67 in the tumor tissues of mice injected with cervical squamous cell carcinoma cells [28]. In this study, silencing EP300 in HBV‐positive HCC cell lines reduced tumor growth in vitro and in vivo, accompanied by lower HBeAg and HBsAg concentrations and Ki67 positivity. However, in vivo, fluorescence imaging should be considered in our following studies to validate the conclusion.

EP300 showed remarkable selectivity for sites on H3, targeting K18 and K27, while not influencing neighboring sites at K14 and K23 [29]. It has been reported by Ibrahim et al. that treating HepG2 cells with histone deacetylase inhibitors enhanced H3K27ac accumulation at the transcription start site of hNTCP, significantly increasing hNTCP mRNA and protein levels, and rendering the cells susceptible to HBV infection [30]. To explore the downstream effects of EP300 in the malignant behavior of HBV‐positive HCC cells, bioinformatics analyses identified MCM8 as a promising target. MCM8 belongs to the MCM protein family, which participates in various DNA‐related functions and disorders, such as DNA replication, meiosis, homologous recombination, and mismatch repair [31]. MCM8 upregulation was related to advanced tumor grade and lymph node metastasis, and indicated poor prognosis in lung cancer, bladder cancer, as well as gastric cancer [32, 33, 34]. The role of MCM8 in promoting various human cancer cell phenotypes has been highlighted in colorectal cancer [35] and osteosarcoma [36]. While increased MCM8 expression has been correlated with poor prognosis and immune cell infiltration in HCC [37], its exact functions in disease progression remain unclear. Interestingly, increased stability of MCM2 and MCM5 transcripts has been found to promote cell cycle progression and HBV‐related hepatocellular tumorigenesis [38]. In contrast, inhibiting MCM7 protein has been found to reduce HBV activity in SNU‐398 cells [39]. Here, we found increased MCM8 expression in HBV‐positive tumors and HCC cells, showing a consistent trend with existing literature and public datasets. Furthermore, *EP300* silencing in HBV‐positive HCC cells led to decreased *MCM8* expression by reducing H3K27ac modification. Restoring *MCM8* expression in these cells rescued proliferation, migration, invasion, and apoptosis resistance suppressed by *EP300* silencing. This ample evidence demonstrates the involvement of *MCM8* in the *EP300*‐mediated malignant phenotype of HBV‐positive HCC cells.

## 5 Conclusion

**FIGURE 7:**
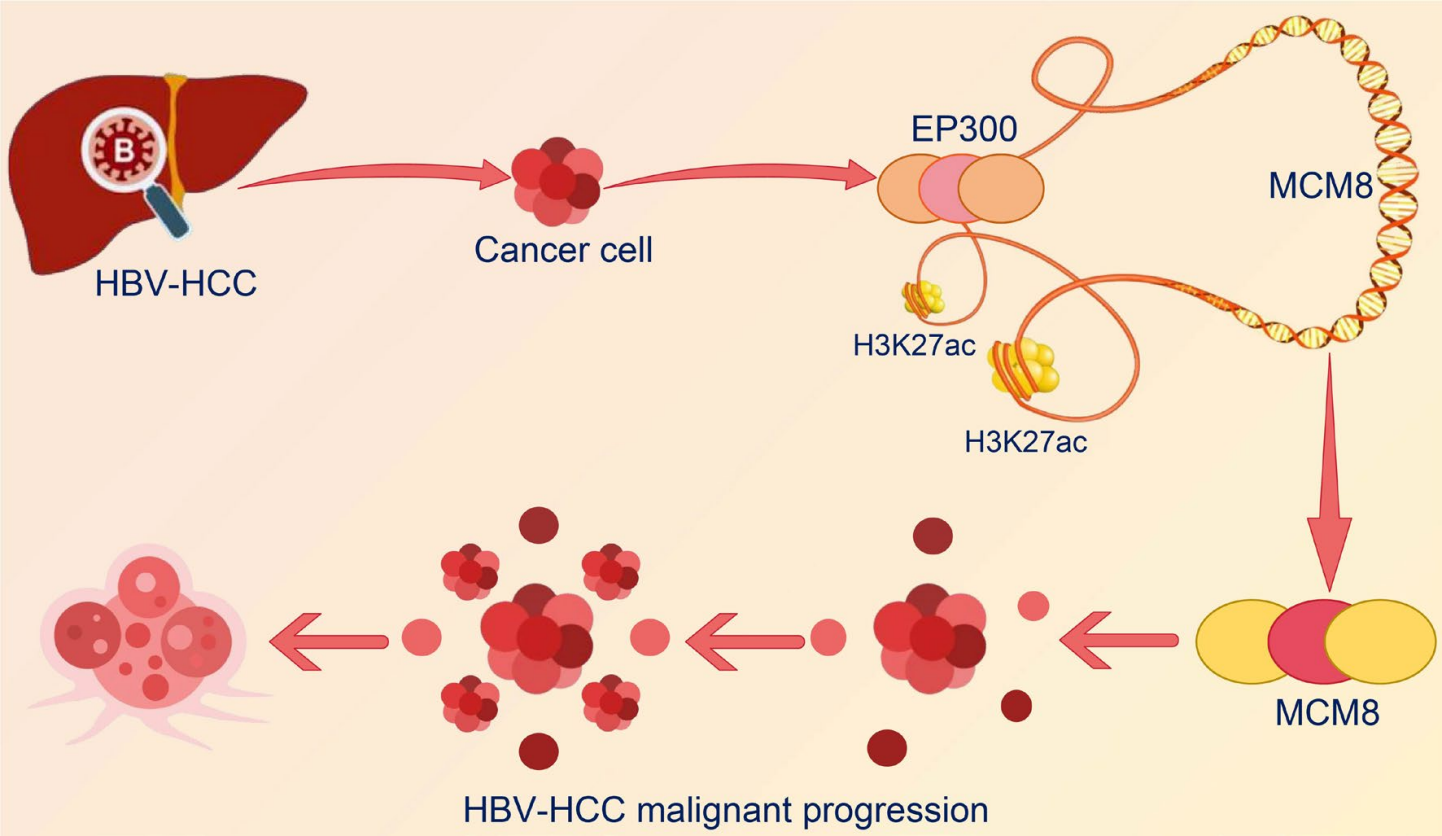
Schematic of the role of EP300 in mediating H3K27ac modification of the MCM8 promoter in HBV‐positive HCC cells.

In summary, this study demonstrates that *EP300* is upregulated in HBV‐positive HCC, which aggravates the malignant properties of cells by upregulating *MCM8* through acetylation modification (Figure 7). Therefore, EP300 and MCM8 may serve as promising therapeutic targets for HBV infection and the associated HCC progression.

## Conflicts of Interest

The authors declare no conflicts of interest.

## Supporting information


**Table S1.** Clinicopathologic features of patients with HCC.

## Data Availability

The data that support the findings of this study are available from the corresponding author upon reasonable request.
