# Supplementary material for: EP300 Modulates MCM8 Transcription and Augments the Malignant Phenotype of Hepatitis B Virus–Positive Hepatocellular Carcinoma Cells
Source: Kaohsiung J Med Sci. 2025 Mar 17;41(6):e70006. doi: 10.1002/kjm2.70006 (PMC12199581; doi:10.1002/kjm2.70006)
Supplement: Supplementary file 1 — Table S1. Clinicopathologic features of patients with HCC. [file KJM2-41-e70006-s001.docx]

Supplementary table 1 Clinicopathologic features of patients with HCC

| Number | Age (years) | ALT (U/L) | FIB-4 | HBsAg (Log U/mL) | HBeAg (PEIU/mL) | HBV DNA load (Log U/mL) |
| --- | --- | --- | --- | --- | --- | --- |
| 1 | 32 | 231.48 | 0.68 | 3.99 | 620.52 | 6.79 |
| 2 | 43 | 261.2 | 0.95 | 3.67 | 1190.34 | 7.47 |
| 3 | 27 | 154.59 | 1.45 | 4.77 | 496.13 | 7.5 |
| 4 | 39 | 231.77 | 0.89 | 4.92 | 1119.59 | 7.01 |
| 5 | 35 | 301.3 | 1.04 | 3.82 | 1062.05 | 6.29 |
| 6 | 47 | 231.98 | 2.17 | 4.09 | 856.25 | 8.61 |
| 7 | 52 | 205.04 | 0.9 | 3.69 | 1048.77 | 8.13 |
| 8 | 24 | 280.18 | 0.62 | 2.53 | 815.74 | 6.82 |
| 9 | 36 | 254.15 | 0.78 | 3.6 | 558.03 | 8.82 |
| 10 | 33 | 312.64 | 2.09 | 3.39 | 1176.28 | 5.44 |
| 11 | 46 | 208.05 | 2.74 | 4.56 | 866.03 | 8.77 |
| 12 | 38 | 338.22 | 1.78 | 2.46 | 826.02 | 8.33 |
| 13 | 35 | 206.19 | 0.74 | 3.54 | 855.13 | 5.39 |
| 14 | 37 | 243.36 | 0.47 | 2.83 | 792.83 | 5.22 |
| 15 | 48 | 123.32 | 1.36 | 3.67 | 633.81 | 6.87 |
| 16 | 41 | 326.19 | 0.89 | 3.97 | 926.53 | 7.4 |
| 17 | 30 | 147.24 | 2.32 | 2.49 | 1058.74 | 8.09 |
| 18 | 26 | 204.07 | 0.59 | 4.52 | 985.13 | 7.88 |
| 19 | 58 | 250.61 | 2.84 | 3.95 | 1150.64 | 6.45 |
| 20 | 46 | 257.36 | 1.81 | 3.92 | 582.22 | 6.13 |
| 21 | 37 | 309.08 | 0.57 | 3.54 | 861.07 | 8.63 |
| 22 | 49 | 165.54 | 0.8 | 4.57 | 943.06 | 8.03 |
| 23 | 51 | 222.48 | 2.18 | 2.61 | 740.85 | 8.81 |
| 24 | 60 | 243.1 | 2.18 | 4.18 | 1372.33 | 7.56 |

Note: HCC, hepatocellular carcinoma; ALT, alanine aminotransferase; FIB-4, fibrosis-4 index; HBsAg, hepatitis B surface antigen; HBeAg, hepatitis B virus early antigen.
